# The incremental significance of heart rate recovery as a predictor during exercise-stress myocardial perfusion SPECT imaging in individuals with suspected coronary artery disease

**DOI:** 10.3389/fcvm.2023.1082019

**Published:** 2023-03-23

**Authors:** Shuai Yang, Rui Xi, Bing-Bing Li, Xin-Chao Wang, Li-Wei Song, Tian-Xiong Ji, Hui-Zhu Ma, Hai-Li Lu, Jing-Ying Zhang, Si-Jin Li, Zhi-Fang Wu

**Affiliations:** ^1^ Department of Nuclear Medicine, First Hospital of Shanxi Medical University, Taiyuan, China; ^2^ Collaborative Innovation Center for Molecular Imaging of Precision Medicine, First Hospital of Shanxi Medical University, Taiyuan, China; ^3^ School of Public Health, Shanxi Medical University, Taiyuan, China; ^4^ Department of General Medical Dept, First Hospital of Shanxi Medical University, Taiyuan, China; ^5^ Key Laboratory of Cellular Physiology, Ministry of Education, Shanxi Medical University, Taiyuan, China

**Keywords:** heart rate recovery, major adverse cardiac events, prognostic, myocardial perfusion imaging (MPI), SPECT, coronary artery disease

## Abstract

**Background:**

Major adverse cardiac events (MACE) are more likely to occur when abnormal heart rate recovery (HRR). This study aimed to assess the incremental predictive significance of HRR over exercise stress myocardial perfusion single-photon emission computed tomography (MPS) results for MACE in individuals with suspected coronary artery disease (CAD).

**Methods:**

Between January 2014 and December 2017, we continually gathered data on 595 patients with suspected CAD who received cycling exercise stress MPS. HRR at 1, 2, 3, and 4 min were used as study variables to obtain the optimal cut-off values of HRR for MACE. The difference between the peak heart rate achieved during exercise and the heart rate at 1, 2, 3, and 4 min was used to calculate the HRR, as shown in HRR3. Heart rate variations between two locations in time, such as HRR_2 min−1 min_, were used to establish the slope of HRR. All patients were followed for a minimum of 4 years, with MACE as the follow-up goal. The associations between HRR and MACE were assessed using Cox proportional hazards analyses.

**Results:**

Patients with MACE were older (*P* = 0.001), and they also had higher rates of hypertension, dyslipidemia, diabetes, abnormal MPS findings (SSS ≥ 5%), medication history (all *P* < 0.001), and lower HRR values (all *P* < 0.01). Patients with and without MACE did not significantly vary in their HRR_4 min−3 min_. The optimal cut-off of HRR1, 2, and 3 combined with SSS can stratify the risk of MACE in people with suspected CAD (all *P* < 0.001). HRR 1, 2, and 3 and its slope were linked to MACE in multivariate analysis, where HRR3 was the most significant risk predictor. With a global X^2^ increase from 101 to 126 (*P* < 0.0001), HRR3 demonstrated the greatest improvement in the model's predictive capacity, incorporating clinical data and MPS outcomes.

**Conclusions:**

HRR at 3 min has a more excellent incremental prognostic value for predicting MACE in patients with suspected CAD following cycling exercise stress MPS. Therefore, incorporating HRR at 3 min into known predictive models may further improve the risk stratification of the patients.

## 1. Introduction

Accurate risk classification and prognosis evaluation are crucial due to the rising incidence and mortality of coronary artery disease (CAD) (1). For the diagnosis of myocardial ischemia and the prognosis of major adverse cardiac events (MACE) in patients with suspected CAD, myocardial perfusion single-photon emission computed tomography (MPS) is a crucial imaging technique (2, 3). Heart rate recovery (HRR) represents the balance between parasympathetic reactivation and sympathetic regression based on exercise stress testing (ET) (4, 5). The correlation between aberrant HRR and the incidence of MACE in individuals with suspected CAD has been supported by several research (6–10).

There are no established standards for determining the best HRR time point for predicting MACE in individuals with suspected CAD, however. Additionally, it is uncertain whether HRR has additional predictive value for exercise stress MPS. To predict MACE in patients with suspected CAD undergoing exercise stress MPS, this research sought to retrospectively explore the predictive usefulness of HRR at various time points about other clinical factors and MPS outcomes.

## 2. Materials and methods

### 2.1. Study population

At the First Hospital of Shanxi Medical University (China), we analyzed 1,090 patients with known or suspected CAD who were referred for clinically indicated MPS with cycling exercise stress between January 2014 and December 2017. The following was taken into account as exclusion standards: (1) Patients with known CAD (known coronary stenosis ≥50%), a history of myocardial infarction (MI), percutaneous coronary intervention (PCI), or coronary artery bypass graft (CABG), early revascularization (coronary angioplasty or CABG surgery occurring <90 days after MPS), diagnosed heart failure (HF), significant cardiac valve disease, severe nonischemic cardiomyopathy, congenital heart disease, pacemaker patients, or any condition that could affect short-term prognosis. (2) those in whom ET duration was <6 min. (3) incomplete exercise stress testing or SPECT/CT data. We did not exclude but adjusted in the multivariable models with medications that affect HR, such as calcium channel blockers (including non-dihydropyridines) or β-blockers (12.8% and 4.8%, respectively).

**Figure 1:**
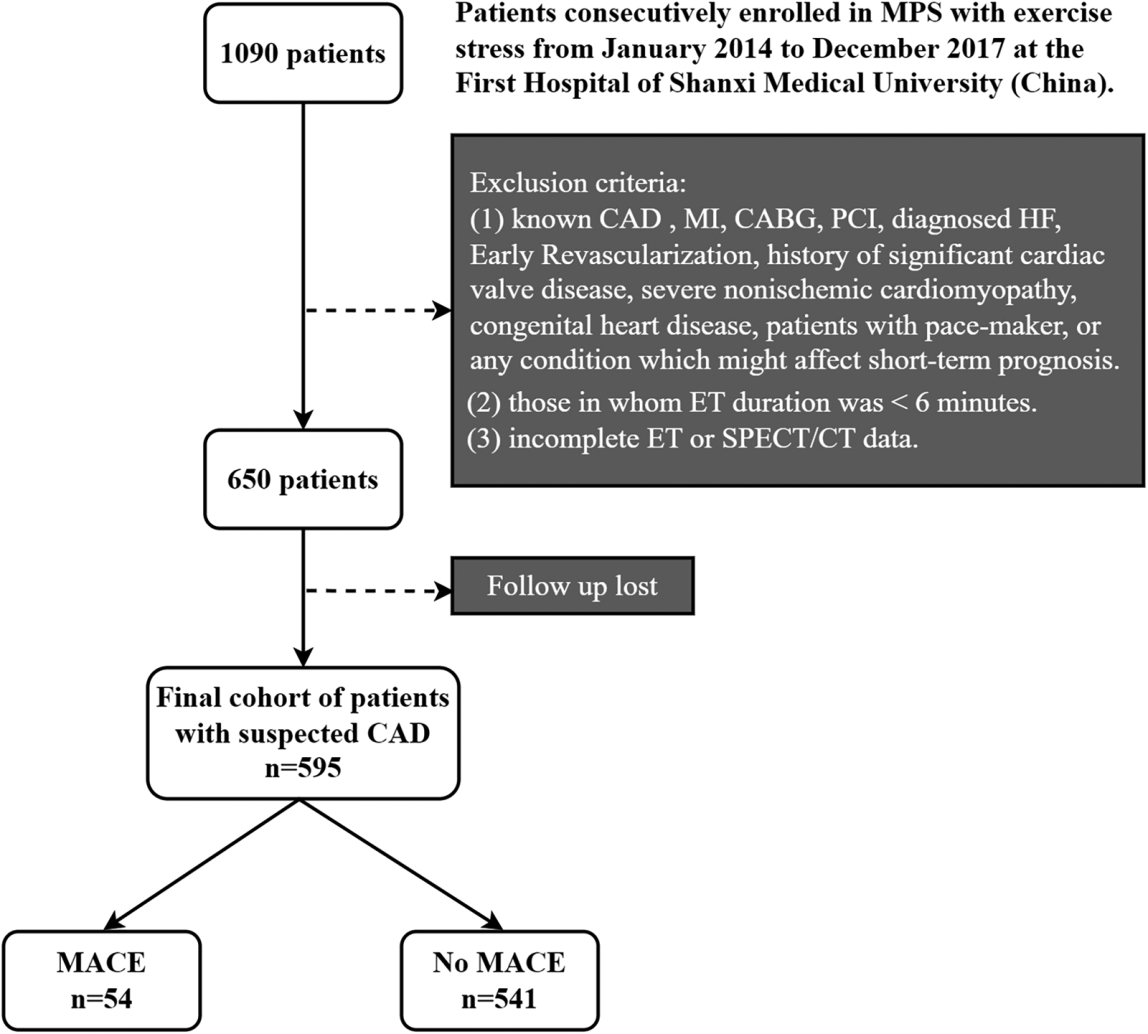
Flowchart of the study cohort. MPS, myocardial perfusion single-photon emission computed tomography; CAD, coronary artery disease; SPECT, single-photon emission computed tomography; ET, exercise test; MACE, major adverse cardiovascular events; MI, history of myocardial infarction; CABG; coronary artery bypass graft; PCI, percutaneous coronary intervention; HF, heart failure.

Out of 650 patients who met all the requirements, 595 (91.5% of the total) had their follow-ups completed. Figure 1 displays the flowchart for the study cohort. The First Hospital of Shanxi Medical University's ethical committee accepted the research protocol, which adhered to the Declaration of Helsinki.

### 2.2. Clinical data

Age, gender, body mass index (BMI), family history of CAD, hypertension, diabetes, hyperlipidemia, and medication history were the demographic and cardiovascular risk variables extracted from electronic medical records. Blood pressure >140/90 mmHg or using antihypertensive medication were both considered to be indicators of hypertension. Patients were deemed to have diabetes if they had previously been diagnosed with it or were using hypoglycemic drugs. Hyperlipidemia was defined as having a known history of the condition or being treated with a lipid-lowering drug. Prior or ongoing tobacco consumption was referred to as smoking history. A first-degree relative's diagnosis of CAD before or at the age of 55 was considered a diagnostic of early CAD (11).

### 2.3. Exercise and image protocol

According to the recommendations of the American Society of Nuclear Cardiology (ASNC), patients received stress ^99m^Tc-sestamibi gated MPS using the symptom-limited Bruce Cycling protocol (3, 12). All individuals were asked to skip 48 h of taking calcium antagonists and β-blockers, as well as 12 h of taking long-acting nitrates, before testing. Test endpoints included reaching 85% of the maximum predicted heart rate, excessive ST-segment depression >2 mm from baseline, ST elevation >1 mm in leads without diagnostic Q-waves (aside from leads V1 or aVR), drop in systolic blood pressure >10 mmHg from baseline (accompanied by other evidence of ischemia), blood pressure >230/120 mmHg, moderate to severe angina pectoris, fatigue, noticeable dyspnea, dizziness, or clinically important cardiac arrhythmia. Throughout the test, blood pressure, ECG, heart rate, and rhythm were all monitored (12).

Patients exercised for an additional 60 s at their maximum exertion after receiving a dosage of ^99m^Tc-sestamibi (740 ± 74) MBq intravenously. The maximum heart rate attained while exercising was the peak heart rate. When calculating HRR, the difference between the peak heart rate and the heart rate during the recovery period was considered, as in HRR1. During the recovery phase, the measurement time was at least 4 min (12), including active recovery (unloaded pedaling) for the first 20–30 s and passive recovery (cease all activities) for the rest of the time. The posture was half-supine throughout the recovery process. The heart rate difference between two locations in time, such as HRR_2 min−1 min_, was used to establish the slope of HRR (13). Exercise capacity was assessed based on the peak METs achieved. The software Cardiosoft V6.5 (General Electric) calculates METs according to the formula:

METs=[(watt×12)+(bodyweight×3.5)]/(bodyweight×3.5).

Following the tracer injection, image capture occurred 30–60 min later. Utilizing a dual-head 90° gamma camera (Symbia T16, Siemens Medical Systems, Erlangen, Germany), MPS images were obtained using the gated SPECT technique. There was no scatter, or attenuation adjustment applied.

### 2.4. Image analysis

All images were collectively evaluated by two skilled cardiologists. The gated myocardial perfusion pictures were visually interpreted using the 17-segment method in a semi-quantitative manner (3). From normal (score = 0) to lacking perfusion (score = 4), each cardiac section was graded. The scores from the 17 stress image segments were added to get the summed stress score (SSS), representing the entire aberrant myocardium (i.e., necrotic and ischemic tissue). Each reader scored based on qualitative visual evaluation and quantitative perfusion data. By computing the ratio between SSS and its maximum potential score (68 score), the total cardiac defect extension % was created using SSS (%). Myocardial SSS ≥ 5% indicated a possible ischemia reaction (14). Using the QPS and QGS 2009 software packages (Cedars-Sinai Medical Center, Los Angeles, CA, United States), ventricular function variables such as left ventricular ejection fraction (LVEF), end-systolic, and end-diastolic volumes (ESV and EDV, respectively), and phase analysis variables such as phase bandwidth, phase SD, and entropy, were automatically calculated.

### 2.5. Study end points

The endpoint was MACE, which comprised all-cause death, nonfatal MI, unstable angina (UA), or late (>90 days after SPECT imaging) coronary revascularization (PCI or CABG) (15). By checking the patients' clinical records and calling the patients, their family members, or the doctor who referred them, follow-up was carried out. In December 2021, all follow-ups were completed. The follow-up period was calculated from the MPS examination date to (the first) MACE or the follow-up date (15).

### 2.6. Statistical analysis

Numbers and percentages are used to represent categorical variables. The mean ± standard deviation (SD) or median values for continuous variables are shown (interquartile range). Categorical data were compared using the *χ*^2^ test, while continuous variables were compared using the appropriate Mann–Whitney *U* or student *t*-test. The Youden index was used to determine the HRR cutoff values that were most effective in forecasting MACE. The main result of MACE was evaluated using log-rank tests on Kaplan–Meier survival curves stratified by SSS and HRR factors.

To comprehensively investigate the predictive power of HRR on MACE at various time points, univariable and multivariable Cox regression models were used. The multivariable model considered variables that have been shown to have statistical significance in univariable analysis (*P* < 0.05) or clinical significance. Separate Cox proportional hazard models were used to determine the hazard ratios (HRs) and 95% confidence intervals (CIs) for each HRR. Using the Schoenfeld residuals test, the proportional hazard assumption was put to the test. None of the Cox model's included variables resulted in the proportionate hazard assumption being rejected. Based on the likelihood ratio test, we further looked for possible statistically significant interactions. To assess the additional value of MPS findings in comparison to clinical features alone and HRR in comparison to clinical characteristics plus MPS results, global *χ*^2^ analyses were performed using Cox models and a likelihood ratios test. Statistical significance was defined as a 2-sided *P* < 0.05. All analyses were carried out using R version 4.1.1 and SPSS v25.0 (SPSS Inc., Chicago, IL, United States).

## 3. Results

### 3.1. Patient features and the results

595 patients made up the final research population. Table 1 displays the baseline characteristics of these individuals. Patients with MACE had were older (51.7 vs. 57.1 years, *P* = 0.001) and lower Phase SD than patients without MACE. They also had larger percentages of combined hypertension, dyslipidemia, diabetes, SSS ≥ 5%, maximum heart rate, maximal systolic blood pressure, and medication history (exclude ACEI/ARB, *P* = 0.549). It took an average of 5.4 ± 1.2 years to follow up. During follow-up, 54 (9.1%) patients had MACE, which included the initial incident of 8 fatalities, 4 nonfatal MI, 29 UA hospitalizations, and 13 late revascularizations.

**Table 1:** Clinical characteristics and imaging findings in 595 patients with suspected CAD undergoing exercise stress-MPS.

Variables	Overall (*N* = 595)	No MACE (*N* = 541)	MACE (*N* = 54)	*P*-value
Age, years	52.2 ± 11.2	51.7 ± 11.1	57.1 ± 10.1	0.001
Male, *n* (%)	262 (44.0)	233 (43.1)	29 (53.7)	0.175
BMI, kg/m^2^	24.5 (22.6, 26.6)	24.5 (22.5, 26.6)	25.4 (23.3, 26.5)	0.184
Hypertension, *n* (%)	191 (32.1)	158 (29.2)	33 (61.1)	<0.001
Hypercholesterolemia, *n* (%)	123 (20.7)	94 (17.4)	29 (53.7)	<0.001
Diabetes, *n* (%)	47 (7.9)	35 (6.5)	12 (22.2)	<0.001
Current smoker, *n* (%)	130 (21.8)	113 (20.9)	17 (31.5)	0.104
Family history of CAD, *n* (%)	59 (9.9)	50 (9.2)	9 (16.7)	0.133
EDV, ml	64.0 (52.0, 78.0)	64.0 (52.0, 78.0)	62.0 (53.2, 79.8)	0.837
ESV, ml	27.0 (20.0, 37.0)	27.0 (20.0, 37.0)	27.5 (21.0, 38.8)	0.749
LVEF (%)	57.0 (52.0, 63.0)	57.0 (52.0, 63.0)	57.0 (50.2, 60.8)	0.427
Bandwidth (°)	42.0 (30.0, 54.0)	42.0 (30.0, 54.0)	36.0 (30.0, 48.0)	0.098
Mean (°)	136.6 (127.2, 147.0)	136.2 (127.0, 146.6)	139.3 (128.0, 148.0)	0.392
Phase SD (°)	14.6 (8.7, 22.6)	15.2 (9.0, 23.0)	10.4 (7.6, 19.4)	0.018
Entropy (%)	39.0 (35.0, 45.0)	40.0 (35.0, 45.0)	39.0 (34.2, 43.8)	0.422
SSS ≥ 5 (%)	49 (8.2)	36 (6.7)	13 (24.1)	<0.001
Maximum systolic BP, mmHg	168 (152, 182)	167.0 (151, 181)	174.0 (164, 190)	0.005
Maximum diastolic BP, mmHg	85 (77, 94)	85.0 (76, 94)	86.5 (79, 96)	0.305
METs	5.4 (4.5, 6.3)	5.5 (4.6, 6.3)	5.1 (4.2, 5.8)	0.074
Resting HR (bpm)	74 (65, 83)	74.0 (65, 83)	74 (68, 86)	0.380
Maximum HR (bpm)	144 (133, 151)	144 (134, 151)	136 (123, 146)	<0.001
Medication, *n* (%)	157 (26.4)	127 (23.5)	30 (55.6)	<0.001
β-Blocker	29 (4.8)	22 (4.1)	7 (13.0)	0.010
CCB	76 (12.8)	63 (11.6)	13 (24.1)	0.009
ACEI or ARB	48 (8.1)	42 (7.8)	6 (11.1)	0.549
Statin	56 (9.4)	41 (7.6)	15 (27.8)	<0.001
Hypoglycemic	28 (4.7)	20 (3.7)	8 (14.8)	<0.001
Platelet inhibitor	20 (3.4)	14 (2.6)	6 (11.1)	0.004

Values are shown as mean ± SD, median [25th–75th percentiles], or number (%) of patients. BMI, indicates body mass index; CAD, coronary artery disease; MACE, major adverse cardiac events; HR, heart rate; BP, blood pressure; SSS, summed stress score; LVEF, left ventricle ejection fraction; METs, metabolic equivalents; CCB, calcium channel blocker; ACEI, angiotensin-converting enzyme inhibitor; ARB, angiotensin receptor blocker.

**Figure 2:**
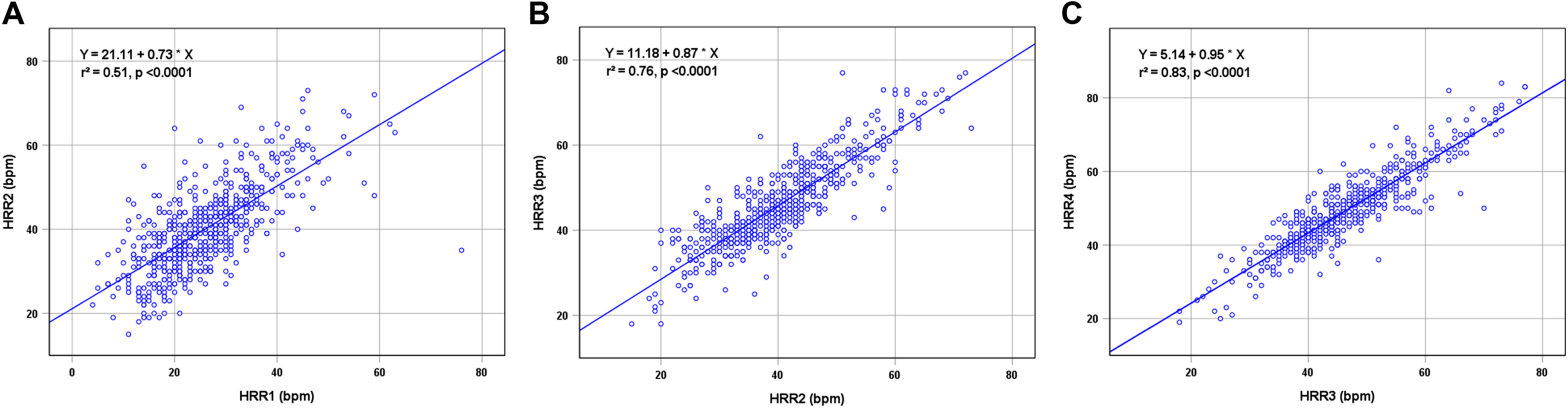
Correlations between HRR1 and HRR2 (**A**), HRR2 and HRR3 (**B**), and HRR3 and HRR4 (**C**). HRR, heart rate recovery.

Table 2 displays the findings for HRR and HRR slope. The HRR at each time, HRR_2 min−1 min_ and HRR_3 min−2 min_ values for the patients with MACE were substantially lower (all *P* < 0.01), but HRR_4 min−3 min_ did not differ between MACE and without MACE (*P* = 0.576). The correlation of HRR is presented in Figure 2. All adjacent HRR positively correlated with each other, with HRR3 and HRR4 showing the strongest correlation (*r*^2^ = 0.51 for HRR1 and HRR2, *r*^2^ = 0.76 for HRR2 and HRR3, *r*^2^ = 0.83 for HRR3 and HRR4). Taking into account the fact that patients with and without MACE did not have any discernible changes in HRR_4 min−3 min_ and the fact that HRR3 and HRR4 have a high association (Figure 2), this study will not discuss the prognostic value of HRR4 for MACE. In addition, HRR at all time points was not correlated with SSS (*P* > 0.05).

**Table 2:** Heart rate recovery and slope characteristics.

Variables	Overall (*N* = 595)	No MACE (*N* = 541)	MACE (*N* = 54)	*P*-value
**Heart rate recovery (bpm)**				
HRR1	25 (20, 31)	26 (20, 32)	21 (18, 26)	0.001
HRR2	40 (34, 46)	40 (34, 46)	33 (28, 40)	<0.001
HRR3	45 (39, 52)	45 (40, 52)	37 (32, 43)	<0.001
HRR4	48 (42, 54)	49 (43, 55)	39 (35, 46)	<0.001
**Heart rate recovery slope**				
HRR_2 min−1 min_	14.0 (10.0, 18.0)	14.0 (10.0, 18.5)	11.5 (7.8, 15.0)	0.003
HRR_3 min−2 min_	5.0 (3.0, 9.0)	6.0 (3.0, 9.0)	3.0 (2.0, 7.0)	0.003
HRR_4 min−3 min_	3.0 (1.0, 5.0)	3.0 (1.0, 5.0)	3.0 (1.0, 5.0)	0.576

Values are median [25th–75th percentiles]. HRR, heart rate recovery.

### 3.2. Ideal cutoff values for Kaplan–Meier and HRR analysis

**Figure 3:**
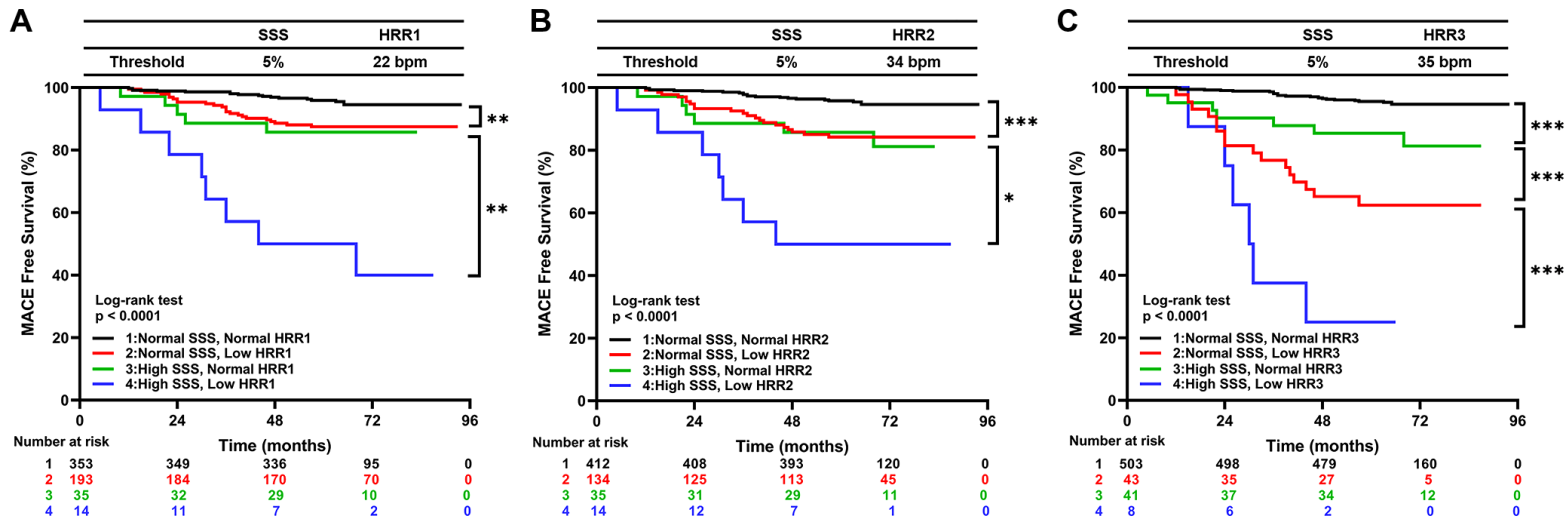
Kaplan–Meier curves for MACE by the groups based on SSS and HRR1 (**A**), SSS and HRR2 (**B**), and SSS and HRR3 (**C**). MACE, major adverse cardiac events; HRR, heart rate recovery; SSS, summed stress score. *p < 0.05, **p < 0.01, ***p < 0.001.

For HRR1, HRR2, and HRR3, the ideal cutoff values were defined as 22 bpm, 34 bpm, and 35 bpm, respectively. The appropriate cutoff value of HRR1, 2, and 3 and the SSS abnormalities were used to build the Kaplan-Meier survival curves for MACE (Figures 3A–C). MACE was additively predicted by adding each variable to the others (all *P* < 0.0001; Figure 3).

### 3.3. Cox regression analysis

In Table S1, it is shown how demographic and initial clinical factors relate to the risk of MACE. The association between HRR and MACE is presented in Table 3. HRR1, 2, and 3 and HRR_2 min−1 min_, and HRR_3 min−2 min_ were associated with MACE in univariate and multivariable Cox regression analyses. Table 3 shows a time trend for hazard ratio in the multivariable models, with hazard ratio decreasing for every one-minute increase in HRR, where HRR3 had the lowest hazard ratio and was the most significant risk predictor.

**Table 3:** Unadjusted and adjusted hazard ratios for MACE by HRR or HRR slope.

Model	Univariable analysis		Multivariable analysis	
	Hazard ratio (95% CI)	*P*-value	Hazard ratio (95% CI)	*P*-value
**HRR1 (bpm)**				
Continuous	0.951 (0.921–0.982)	0.002	0.951 (0.921–0.983)	0.003
HRR1 ≥ 22	0.427 (0.248–0.735)	0.002	0.472 (0.273–0.816)	0.007
**HRR2 (bpm)**				
Continuous	0.923 (0.894–0.954)	<0.0001	0.928 (0.899–0.958)	<0.0001
HRR2 ≥ 34	0.265 (0.154–0.457)	<0.0001	0.279 (0.162–0.482)	<0.0001
**HRR3 (bpm)**				
Continuous	0.888 (0.859–0.919)	<0.0001	0.902 (0.872–0.933)	<0.0001
HRR3 ≥ 35	0.117 (0.068–0.200)	<0.0001	0.119 (0.068–0.208)	<0.0001
**HRR slope (bpm)**				
HRR_2 min−1 min_	0.963 (0.936–0.992)	0.011	0.963 (0.932–0.995)	0.025
HRR_3 min−2 min_	0.920 (0.872–0.970)	0.002	0.922 (0.871–0.976)	0.005

Hazard ratios for major adverse cardiac events (MACE) were estimated using a Cox proportional hazard model. All models were adjusted with cardiac risk factors (age, gender, hypertension, hyperlipidemia, diabetes), exercise stress MPS variables (metabolic equivalents, resting heart rate, maximum systolic blood pressure, left ventricle ejection fraction, summed stress score ≥5%, stress phase SD), and medication history (β-Blocker, calcium channel blocker, statin, hypoglycemic, platelet inhibitor). Hazard ratio and confidence interval (CI) are shown continuous and dichotomous HRR. CI, confidence interval; HRR, heart rate recovery; N.S, not-significant.

We investigated whether HRR2 could explain the link between HRR3 and MACE by fitting HRR2 and HRR_3 min−2 min_ in a multivariate model. The results showed that both HRR2 (hazard ratio, 0.905; CI, 0.874–0.937 [*P* = 2.76 * 10^−08^]) and HRR_3 min−2 min_ (hazard ratio, 0.890; CI, 0.841–0.941 [*P* = 4.3 * 10^−05^]) were significantly associated with MACE. This result confirms that HRR2 and HRR_3 min−2 min_ contribute to the predictive value of HRR3 for MACE. Interactions of HRR3 with β-blockers, CCBs and statins are shown in Table S2. In the multivariate model, a significant interaction was found between HRR3 ≥ 35 and β-blockers (Table S2, *P*-value for interaction =0.013), whereas there was no interaction when treated as a continuous variable. There was no significant interaction between CCBs and statins, regardless of whether HRR3 was considered a continuous or dichotomous variable. Therefore, continuous HRR3 was included in the model to assess its incremental value.

### 3.4. Incremental value of HRR

**Figure 4:**
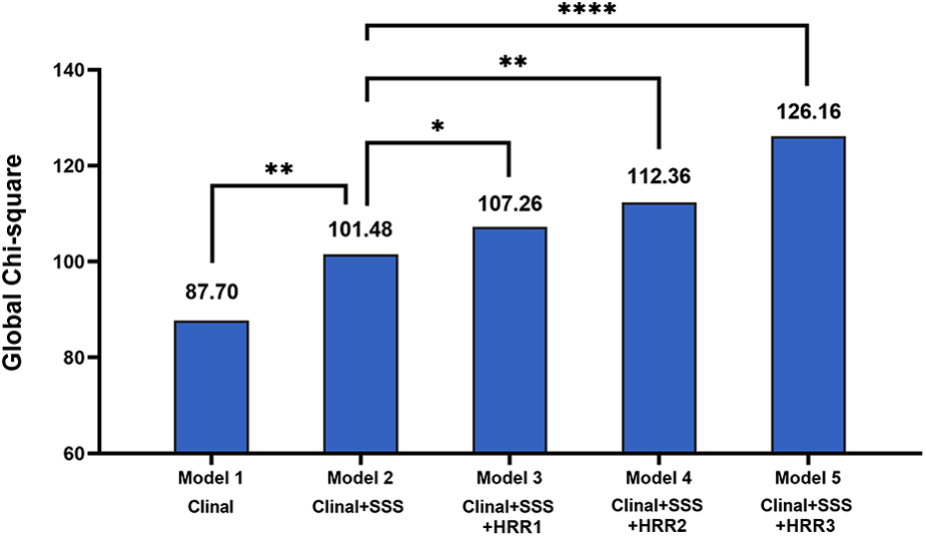
Incremental value of HRR variables for prediction of MACE beyond clinal data and SSS. MACE, major adverse cardiac events; HRR, heart rate recovery; SSS, summed stress score. *p < 0.05, **p < 0.01, ****p < 0.0001.

The global *χ*^2^ findings for the MACE prediction are shown in Figure 4. Compared to model 1 (clinical data alone, *P* = 0.009), the global *χ*^2^ for model 2 (clinical data plus SSS) dramatically increased. Additionally, global *χ*^2^ for the models that included clinal data, SSS, and HRR variables (model 3: clinal data + SSS + HRR1, model 4: clinal data + SSS + HRR2, model 5: clinal data + SSS + HRR3) were significantly higher than those for model 2 (*P* = 0.042 for model 3, *P* = 0.001 for model 4, *P* < 0.0001 for model 5, Figure 4), demonstrating that HRR variables increase the discriminatory power.

## 5. Discussion

For individuals with known or suspected CAD, studies of HRR acquired from exercise stress MPS for predicting death have been published (16). To predict MACE in patients with suspected CAD, this research examined the incremental value of HRR for the first time at various time points (1–4 min) during exercise stress MPS. The following are the study's key findings: (1) HRR at 1–3 min predicted MACE with the recovery protocol after cycling ET as a combination of active no-load and passive rest recovery in the half-supine position, this agrees with the findings of earlier research (6, 8, 10, 16), (2) Our study found that HRR3 had the strongest predictive power and incremental prognostic value compared to HRR measured at earlier time points and that 35 bpm for HRR3 was the optimal cut-off value for predicting MACE. However, this differs from the findings of the following scholars. Shetler et al. (17) found that 22 bpm for HRR2 was the optimal cut-off value by extensively studying HRR1 and HRR2 predicting the mortality cut point. In their analysis of computer models of HRR at various time points, Gorelik et al. (13) showed that HRR2 was the most accurate predictor of death. Finally, Gayda et al. (8) considered 46 bpm for HRR3 as the ideal cut-off point for mortality prediction.

The reasons for the discrepancy may be related to the following factors: (1) Different patient demographics and endpoint events were examined in the previous research; the study endpoints were all-cause and cardiac mortality, while MACE was used as the follow-up endpoint in our investigation, which included all patients with suspected CAD. (2) The different ET protocols and recovery positions; The ET protocol described in the former is Treadmill testing with a supine recovery position, whereas our ET protocol is Cycling testing with a half-supine recovery position. Previous studies have found that HRR in the seated position is significantly slower than in the supine or elevated leg position (18). (3) The type of recovery varies; there is a stepwise slowing of HRR from passive (complete resting) to active recovery (maintaining central command and mechanoreceptor action, like unloaded cycling) (19, 20). (4) Differences in exercise capacity; a review of the data from this study revealed that the overall exercise capacity of the patients was weak, with METs of 5.47 ± 1.32. Previous studies have suggested that HRR2 and HRR3 are influenced by the intensity of exercise (21, 22). In addition, exercise-induced accumulation of muscle metabolites and uncontrolled thermoregulation can mediate cardiac sympathetic nerve activity disturbances, ultimately leading to HRR abnormalities (13, 21). However, a recent study found that the predictive value of HRR2 was independent of METs (9), which is consistent with our findings. Therefore, it is more reasonable to explore the optimal time point for HRR to predict endpoint events, considering the study protocol, population, and other conditions.

Although some previous studies have reported that β-blockers, CCBs, and statins affect HRR (23), our study found no significant effect of these drugs on the prognostic value of HRR, which is consistent with the results of several previous studies (17, 24). It is worth noting that β-blockers had a significant interaction with HRR3 ≥ 35 in the present study, but this was not the case when HRR was a continuous variable.

### 5.1. Limitations

Consider some of our study's shortcomings. First, this is a single-center retrospective study, which makes it challenging to obtain a comprehensive set of biomarker data. Second, the low proportion of patients using β-blockers in our milder cohort without severe CAD may have influenced the relationship between such drugs and the prognostic value of HRR, and prospective and large cohort studies are needed to elucidate the possible implications. Third, resting MPS was not included in this study, but current guidelines (3) state that resting MPS may be optionally ignored when patients have negative stress MPS results. Also, some studies have concluded that when stress MPS is negative, combined resting MPS or not has no effect on predicting mortality (25). Therefore, rather than focusing on reversible myocardial ischemia, this research examined the incremental predictive significance of several time points HRR for clinical information and myocardial perfusion deficits in patients with suspected CAD. Finally, to avoid overestimating the prognostic value of clinical and imaging models, we employed continuous variables at various HRR time points to evaluate the additional prognostic value for their inclusion (26). However, in systematic studies, the analysis is often performed using categorized HRR for easier prognostic assessment.

## 6. Conclusions

We provide unique data that links HRR at various time points during cycling exercise testing to MACE in patients with suspected CAD and demonstrate that HRR at 3 min has a stronger incremental value in predicting MACE in patients with suspected CAD compared to HRR at 1 and 2 min for exercise stress MPS. Therefore, assessing such parameters could further improve the risk stratification of patients during the MPS scan.

## Data Availability

The original contributions presented in the study are included in the article/**Supplementary Material**, further inquiries can be directed to the corresponding author/s.
