# Supplementary material for: The incremental significance of heart rate recovery as a predictor during exercise-stress myocardial perfusion SPECT imaging in individuals with suspected coronary artery disease
Source: Front Cardiovasc Med. 2023 Mar 23;10:1082019. doi: 10.3389/fcvm.2023.1082019 (PMC10074983; doi:10.3389/fcvm.2023.1082019)
Supplement: Supplementary file 1 [file Table1.docx]

Supplementary Material

# Supplementary Data

**Table S1. Cox Proportional Hazards Association of Patients’ Characteristics with MACE**

| **Model** | **Univariable analysis** | |  | **Multivariable analysis** | |
| --- | --- | --- | --- | --- | --- |
|  | **Hazard ratio (95% CI)** | **P-value** |  | **Hazard ratio (95% CI)** | **P-value** |
| Age | 1.049（1.021-1.078） | < 0.001 |  | N.S |  |
| Gender, male | 0.673（0.394-1.150） | 0.147 |  | N.S |  |
| Hypertension | 3.516（2.034-6.076） | < 0.001 |  | 2.067（1.147-3.726） | 0.016 |
| Hyperlipidemia | 5.064（2.965-8.650） | < 0.001 |  | 2.865（1.558-5.269） | < 0.001 |
| Diabetes | 3.652（1.922-6.939） | < 0.001 |  | N.S |  |
| METs | 0.834（0.676-1.030） | 0.091 |  | N.S |  |
| Resting HR | 1.011（0.991-1.032） | 0.271 |  | N.S |  |
| Maximum systolic BP | 1.016（1.005-1.028） | 0.005 |  | N.S |  |
| LVEF | 0.988（0.956-1.021） | 0.482 |  | N.S |  |
| SSS ≥ 5% | 4.014（2.150-7.493） | < 0.001 |  | 2.802（1.476-5.318） | 0.002 |
| Stress phase SD | 0.963（0.932-0.995） | 0.024 |  | 0.963（0.931-0.997） | 0.033 |
| β-Blocker | 2.983（1.348-6.600） | 0.007 |  | N.S |  |
| CCB | 2.275（1.219-4.246） | 0.010 |  | N.S |  |
| Statin | 0.993（0.960-1.026） | < 0.001 |  | 2.100（1.103-4.000） | 0.024 |
| Hypoglycemic | 3.860（1.821-8.182） | < 0.001 |  | N.S |  |
| Platelet inhibitor | 3.888（1.664-9.087） | 0.002 |  | N.S |  |
| ACEI or ARB | 1.480（0.633-3.458） | 0.366 |  |  |  |
| Current smoking | 1.674（0.943-2.973） | 0.079 |  |  |  |
| Family history of CAD | 1.877（0.918-3.840） | 0.085 |  |  |  |
| Maximum diastolic BP | 1.007（0.992-1.023） | 0.355 |  |  |  |
| Stress bandwidth | 0.989（0.978-1.001） | 0.073 |  |  |  |
| Stress entropy | 0.993（0.960-1.026） | 0.672 |  |  |  |

Abbreviations: CI, confidence interval; CAD, coronary artery disease; BP, blood pressure; METs, metabolic equivalents; HR, heart rate; LVEF, left ventricle ejection fraction; SSS, summed stress score; CCB, Calcium channel blocker; ACEI, angiotensin-converting enzyme inhibitor; ARB, angiotensin receptor blocker; N.S, not-significant.

**Table S2. Interaction of HRR3 with β-Blockers, CCBs, and Statins**

| **Variables** | **Hazard ratio (95% CI)** | **P-value for interaction** |
| --- | --- | --- |
| HRR3 (Continuous) |  |  |
| HRR3 × β-Blocker | N.S | 0.072 |
| HRR3 × CCB | N.S | 0.538 |
| HRR3 × Statin | N.S | 0.092 |
| HRR3 ≥ 35 |  |  |
| HRR3 ≥ 35 × β-Blocker | 1.833（1.136-2.956） | 0.013 |
| HRR3 ≥ 35 × CCB | N.S | 0.450 |
| HRR3 ≥ 35 × Statin | N.S | 0.246 |

Adjusted for cardiac risk factors (age, gender, hypertension, hyperlipidemia, diabetes), exercise stress MPS variables (metabolic equivalents, resting heart rate, maximum systolic blood pressure, left ventricle ejection fraction, summed stress score ≥ 5%, stress phase SD), and medication history (β-Blocker, calcium channel blocker, statin, hypoglycemic, platelet inhibitor). HRR, heart rate recovery; CCB, Calcium channel blocker; CI, confidence interval; N.S, not-significant.
